# Draft Genome Sequence of *Bacillus mesonae* FJAT-13985^T^ (=DSM 25968^T^) for Setting Up Phylogenomics in Genomic Taxonomy of the *Bacillus*-Like Bacteria

**DOI:** 10.1128/genomeA.00575-16

**Published:** 2016-06-16

**Authors:** Guo-hong Liu, Bo Liu, Yu-jing Zhu, Jie-ping Wang, Jian-mei Che, Qian-qian Chen, Zheng Chen

**Affiliations:** Agricultural Bio-Resources Research Institute, Fujian Academy of Agricultural Sciences, Fuzhou, China

## Abstract

*Bacillus mesonae* FJAT-13985^T^ is a Gram-positive, spore-forming, and aerobic bacterium. Here, we report the draft genome sequence of *B. mesonae* FJAT-13985^T^ with 5,807,726 bp, which will provide useful information for setting up phylogenomics in the genomic taxonomy of the *Bacillus*-like bacteria, as well as for the functional gene mining and application of *B. mesonae* FJAT-13985^T^.

## GENOME ANNOUNCEMENT

We isolated the type strain *Bacillus mesonae* FJAT-13985 (=DSM 25968^T^) from the internal tissues of the *Mesona chinensis* root in Fujian Province, China. The bacterium is widely spread in the soil. As a result of the recent decrease in the cost of genomic sequencing, it has been proposed that whole-genome sequencing information be combined with the main phenotypic characteristics as a polyphasic approach strategy (taxonogenomics) to describe new bacterial taxa (1–4). In this study, a high-quality genome sequence of *B. mesonae* FJAT-13985^T^ was sequenced, which would promote research on the genomic taxonomy of the *Bacillus*-like bacteria.

The genome of *B. mesonae* FJAT-13985^T^ was sequenced with massively parallel sequencing (MPS) Illumina technology. Two DNA libraries were constructed: a paired-end library with an insert size of 500 bp, and a mate-pair library with an insert size of 5 kb. The 500-bp library and the 5-kb library were sequenced using an Illumina HiSeq 2500 with a PE125 strategy. Library construction and sequencing were performed at the Beijing Novogene Bioinformatics Technology Co., Ltd. Quality control of both paired-end and mate-pair reads was performed using an in-house program. After this step, Illumina PCR adapter reads and low-quality reads were filtered. The filtered reads were assembled by SOAP*denovo* (5, 6) to generate scaffolds. All reads were used for further gap closure. Through the data assembly, 5,807,726 bp within 2 scaffolds were obtained, and the scaffold *N*_50_ was 5,806,292 bp. The average length of the scaffolds was 2,903,863 bp, and the longest and shortest scaffolds were 5,806,292 bp and 1,434 bp, respectively.

Gene prediction was performed on the *B. mesonae* FJAT-13985^T^ genome assembly by GeneMarkS (7). Transfer RNA (tRNA) genes were predicted with tRNAscan-SE (8), ribosomal RNA (rRNA) genes were predicted with RNAmmer (9), and small RNAs (sRNAs) were predicted by BLAST against the Rfam (10) database. PHAST (11) is used for prophage prediction, and CRISPRFinder (12) is used for clustered regularly interspaced short palindromic repeat (CRISPR) identification. A total of 6,014 genes were predicted, including 5,867 coding sequences (CDSs), 5 sRNAs, 104 tRNAs, and 38 rRNAs. Also, 9 prophage and 7 CRISPR arrays were found in the draft genome. The average DNA G+C content was 42.89%.

### Nucleotide sequence accession numbers.

This whole-genome shotgun project has been deposited at DDBJ/EMBL/GenBank under the accession no. LUUQ00000000. The version described in this paper is version LUUQ00000000.1.
